# Autoantibodies Activating the β2-Adrenergic Receptor Characterize Patients With Primary and Secondary Glaucoma

**DOI:** 10.3389/fimmu.2019.02112

**Published:** 2019-10-01

**Authors:** Bettina Hohberger, Rudolf Kunze, Gerd Wallukat, Katja Kara, Christian Y. Mardin, Robert Lämmer, Ursula Schlötzer-Schrehardt, Sami Hosari, Folkert Horn, Luis Munoz, Martin Herrmann

**Affiliations:** ^1^Department of Ophthalmology, Friedrich-Alexander-University of Erlangen-Nürnberg, Erlangen, Germany; ^2^Science Office, Berlin-Buch, Campus Max Delbrück Center for Molecular Medicine, Berlin, Germany; ^3^Max Delbrück Center for Molecular Medicine, Berlin, Germany; ^4^Department of Internal Medicine III, Institute of Clinical Immunology and Rheumatology, University of Erlangen-Nürnberg, Erlangen, Germany

**Keywords:** autoantibodies, glaucoma, ocular hypertension, β2-adrenergic receptor, primary open-angle glaucoma, secondary open-angle glaucoma

## Abstract

Recently, agonistic autoantibodies (agAAb) activating the β2-adrenergic receptor were detected in primary open-angle glaucoma (POAG) or ocular hypertension (OHT) patients and were linked to intraocular pressure (IOP) (1). The aim of the present study was to quantify β2-agAAb in the sera of glaucoma suspects and patients with primary and secondary glaucoma. Patients with OHT (*n* = 33), pre-perimetric POAG (pre-POAG; *n* = 11), POAG (*n* = 28), and 11 secondary OAG (SOAG) underwent ophthalmological examinations including examinations with Octopus G1 perimetry and morphometry. Twenty-five healthy individuals served as controls. Serum-derived IgG samples were analyzed for β2-agAAb using a functional bioassay. The beat-rate-increase of spontaneously beating cultured neonatal rat cardiomyocytes was monitored with 1.6 beats/15 s as cut-off. None of the sera of normal subjects showed β2-agAAb. In POAG or OHT patients increased beating rates of 4.1 ± 2.2 beats/15 s, and 3.7 ± 2.8 beats/15 s were detected (*p* > 0.05). Glaucoma patients with (POAG) and without perimetric (pre-POAG) defects did not differ (pre-POAG 4.4 ± 2.6 beats/15 s, POAG 4.1 ± 2.0 beats/15 s, *p* > 0.05). Patients with SOAG yielded mean beating rates of 4.7 ± 1.7 beats/15 s (*p* > 0.05). β2-agAAb were seen in 73% of OHT, 82% of pre-POAG, 82% of POAG, and 91% SOAG patients (*p* < 0.001). Clinical data did not correlate with beating rate (*p* > 0.05). The robust β2-agAAb seropositivity in patients with OHT, pre-POAG, POAG, and SOAG suggest a primary common role for β2-agAAb starting early in glaucoma pathophysiology and turned out to be a novel marker identifying all patients with increased IOP independent of glaucoma stage and entity.

## Introduction

The pathophysiology of glaucoma is known to be multifactorial. In addition to oxidative stress (2), vascular dysregulation (3), or morphological alterations of the trabecular meshwork (4), several further factors may be involved. Intraocular pressure (IOP) is the main risk factor for glaucoma. However, patients with glaucoma developed progressive disease despite optimal pharmaceutical and surgical IOP lowering therapies (5). The β2-adrenergic receptors (β2AR) is likely be involved in glaucoma pathogenesis, as topical application of the β-blocker timolol is an important module of anti-glaucomatous therapies. β2AR are to be found on several ocular tissues. β2AR is expressed by cells of the trabecular meshwork and the ciliary body, responsible for outflow and secretion of aqueous humor (AH), respectively (6, 7). Furthermore, microvessels and optic nerve tissue (8–10) reportedly express β2AR and thus influence microcirculation and neurons, respectively.

Recently, we described specific agonistic autoantibodies (agAAb) activating the β2AR (β2-agAAb) in patients with primary open-angle glaucoma (POAG) and ocular hypertension (OHT). We hypothesize that autoimmunity contributes to the etiopathogenesis of glaucoma. Adsorption of β2-agAAb may influence production and outflow of aqueous humor and, consequently, IOP (11).

The exact role of agonistic agAAb activating adrenergic receptors in disease is still elusive. Chagas' disease (12), allergic asthma (13), idiopathic dilated cardiomyopathy (13) in patients with heart failure (14) are associated with agonistic agAAb. Furthermore, in sera of patients, who suffer from Alzheimer's or vascular dementia β2-agAAb have been reported (15). The β2-agAAb, observed in patients with POAG or OHT, were directed against the second extracellular loop of β2AR (peptides 181–187 and 186–192); the major subclass was IgG3. In a principal-of-proof study IOP decreased transiently after unspecific removal by immunoadsorption of IgG, including the potentially pathogenic β2-agAAb (1). These observations lead us to postulate the hypothesis of an involvement of agonistic β2-agAAb in the pathogenesis of glaucoma. The aim of the present study was to investigate the distribution of agonistic β2-agAAb in glaucoma suspects and in patients with primary and secondary open-angle glaucoma.

## Materials and Methods

### Patients

One hundred-eight individuals were recruited from the Department of Ophthalmology and Eye Hospital, Friedrich-Alexander-University Erlangen-Nürnberg [Erlangen Glaucoma Registry, ISSN 2191-5008, CS-2011; NTC00494923 (5)]. We recruited 33 patients with OHT (54.8 ± 12.8 years, 17 female, 16 male), 11 pre-perimetric POAG (58.8 ± 10.9 years, 5 female, 6 male), 28 POAG (62.2 ± 13.7 years, 11 female, 17 male), and 11 SOAG (50.6 ± 12.5 years, 3 female, 8 male; 6 pigmentary glaucoma, PG; 5 Pseudoexfoliation glaucoma, PEXG). Twenty-five healthy subjects served as controls.

The probands underwent a complete ophthalmological examination including slit-lamp biomicroscopy, funduscopy, and white-on-white perimetry (Octopus 500, Interzeag, Schlieren, Switzerland; program G1). IOP was measured by Goldmann applanation tonometry at five times of the day (10.00 a.m., 12.00 a.m., 4.00 p.m., 9.00 p.m., and 12.00 p.m.). The retinal nerve fiber layer thickness, RNFL, was quantified by Spectralis Optical Coherence Tomography (Spectralis® OCT Version 1.9.10.0, Heidelberg Engineering, Heidelberg, Germany). The study protocol was done in accordance with the tenets of the Declaration of Helsinki and was approved by the Local Ethic Committee.

#### Control Subjects

Controls were screened with slit-lamp biomicroscopy, tonometry, fundoscopy, and papillometry for normal values.

#### Ocular Hypertension (OHT) Patients

Patients with OHT had an increased IOP (>21 mmHg, confirmed at least once), yet no sign of glaucomatous optic disc alterations, and normal perimetric white-on-white findings (G1 perimetry).

#### Primary Open-Angle Glaucoma (POAG) Patients

Diagnosis of POAG was based on an open anterior chamber angle, IOP > 21 mmHg (confirmed at least once), and a glaucomatous optic disc, classified after Jonas (16). Additionally, functional perimetric field loss had to be confirmed at least once according to the following criteria:

(I) at least three adjacent test points having a deviation ≥5 dB and with one test point with a deviation >10 dB lower than normal, or (II) at least two adjacent test points with a deviation ≥10 dB, or (III) at least three adjacent test points with a deviation ≥5 dB abutting the nasal horizontal meridian or (IV) a mean visual field defect of >2.6 dB.

#### Secondary Open-Angle Glaucoma (SOAG) Patients

Secondary open-angle glaucoma (SOAG) were classified according to POAG, yet with additional PEX material (PEXG) or pigmentary dispersion (PG). Additionally, SOAG patients displayed loss of the visual field, according to the before mentioned criteria (see POAG).

#### Primary Pre-perimetric Open-Angle Glaucoma (pre-POAG) Patients

Patients with pre-POAG showed the same morphological findings like those with POAG, however, their visual field was (still) normal.

### Affinity Purification of agAAb

Affinity purification of agAAb was done by a biotinylated peptide biotin-AINCYANETCCD corresponding to the second extracellular loop of the β2AR. After incubation of 1 ml IgG with 300 μl of the peptide (100 μg/ml; for 1 h), this procedure was followed by an incubation with streptavidin-coated magnetic particles (Roche, Germany; for 30 min). The separation was performed with a magnetic separator (Dynal, Germany). After washing with PBS, eluation with 3 M KSCN in two 0.5 ml steps was done. Afterwards the agAAb were dialyzed with PBS (48 h, 4°C).

### Cardiomyocyte Bioassay

Cardiac myocytes were prepared from heart ventricle of 1–2 day-old Sprague-Dawley rats (13). By digestion with a 0.25% solution of crude porcine trypsin (Serva, Germany) the myocardial cells were dispersed and suspended in SM20-I medium (Biochrom, Germany), which contained penicillin (Heyl, Germany), 10% heat-inactivated neonatal calf serum (Gibco, Germany), glutamine (Serva, Germany), streptomycin (HEFA Pharma; Germany), hydrocortisone (Merck, Germany), and fluorodeoxyuridine (Serva, Germany). After seeding the cardiomyocytes with a field density of 160.000 cells/cm^2^, the culture medium was refaced after 24 h. The cells were cultured for 3–4 days at 37°C previous to stimulation. Two hours before onset of the experiments, the medium was renewed with fresh culture solution. Target point was the beating rate for 15 s of 7–10 selected cardiomyocte cells or synchronously contracting cell clusters per flask, placed on a heated stage inverted microscope at 37°C (basal contraction rate: 162 ± 4 beats/min). The cardiomyocytes were incubated with the IgG fractions of probands' sera (duration: 60 min; dilution of 1:40). The target was loop II (HWYRATHQEAINCYANETCCDFFTNQ). The two epitopes were AINCYAN (AS181–187) and ANETCCD (AS186–192).

### Statistical Analysis

Statistical analysis was done using SPSS (version 21.0). Data are displayed as mean ± standard deviation and percentages. Mann-Whitney-*U*-tests were calculated. Correlation analyses were performed.

## Results

**Figure 1:**
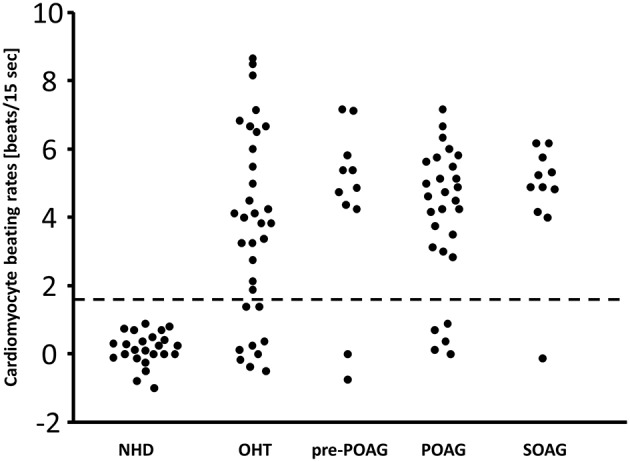
β2-agAAb in patients with OHT, primary, and secondary glaucoma. Cardiomyocyte beating rates [beats/15 s] modulated by IgG from sera of control subjects (normal healthy donor, NHD), patients with ocular hypertension (OHT), pre-perimetric POAG (primary open-angle glaucoma), POAG, and SOAG (secondary open-angle glaucoma) (cut-off 1.6 beats/15 s); cardiomyocyte beating rate is counted as difference of the baseline value (i.e., spontaneously beating rate of cultured cardiomyocytes) and the activated beating rate after addition of the test samples; therefore, some negative values were recorded.

The data of this study is displayed in Figure 1 and Table 1. The beating rate of the cardiomyocytes was 0.15 ± 0.5 beats/15 s in normal subjects. However, patients with glaucoma yielded significant alterations of the cardiomyocytes in the bioassay: patients with OHT and POAG showed increased beating rates of 3.7 ± 2.8 and 4.1 ± 2.2 beats/15 s, respectively. Subgroup analysis for patients with glaucoma with (POAG) and without visual field defect (pre-perimetric POAG) showed mean beating rates of 4.4 ± 2.6 beats/15 s (pre-perimetric POAG) and 4.1 ± 2.0 beats/15 s (POAG). Patients with SOAG yielded mean beating rates of 4.7 ± 1.7 beats/15 s. Subdividing into PEXG and PD showed mean beating rates of 5.2 ± 0.8 beats/15 s and 4.2 ± 2.2 beats/15 s, respectively. No difference in beating rates was seen for group and subgroup analysis (*p* > 0.05, Mann-Whitney-*U*-test).

**Table 1:** Mean beating rates [beats/15 s], absolute number and percentage of β2-agAAb seropositivity of ocular hypertension (OHT), pre-perimetric POAG (pre-perimetric primary open-angle glaucoma), POAG, and SOAG patients in comparison to control subjects. Level of significance was reached for all observed patients' group compared to control.

	**Control**	**OHT**	**Pre-POAG**	**POAG**	**SOAG**
Beats/15 s			4.1 ± 2.2		
	0.15 ± 0.5	3.7 ± 2.8	4.4 ± 2.6	4.1 ± 2.0	4.7 ± 1.7
*p*-Values to control		<0.001^**^	<0.001^**^	<0.001^**^	<0.001^**^
*n*	25	33	11	28	11
Seropositive	0	24	9	23	10
% Seropositive	0	73	82	82	91

** *Significant p < 0.001, Mann-Whitney-U-test*.

With the cut-off value of 1.6 beats/15 s, β2-agAAb were to be observed in 24/33 (73%) of patients with OHT, 9/11 (82%) with pre-perimetric POAG, and 23/28 (82%) with POAG. SOAG patients yielded agAAb in 10/11 (91%). Subdivision into PD and PEXG showed 5/6 (83%) and 5/5 (100%), respectively (Figure 1).

Clinical data of all patients' groups can be seen in Table 2. Beating rates of β2-agAAb seropositive patients showed no correlation with morphometric measurements (RNFL; *p* > 0.05) and functional parameters (mean defect MD; loss variance LV *p* > 0.05). Additionally, no correlation was seen for beating rate of β2-agAAb seropositive patients and maximum IOP of the day of examination (*p* > 0.05). Additionally, not a single significant correlation was to be observed for the stages of glaucoma disease and beating rates (*p* > 0.05). Subgroup analysis yielded no significant correlation of beating rates with MD in POAG, SOAG (*p* > 0.05), respectively. Additionally, beating rates showed no correlation with maximum IOP of the day of examination in β2-agAAb seropositive OHT, pre-perimetric POAG, POAG, and SOAG (*p* > 0.05). RNFL was not correlated with beating rates in β2-agAAb seropositive pre-perimetric POAG, POAG, and SOAG (*p* > 0.05). Thus, the beat rate discriminated between all patients with increased IOP independent of glaucoma stage and entity.

**Table 2:** Clinical data of OHT (ocular hypertension), pre-perimetric POAG (primary open-angle glaucoma), POAG, and SOAG (secondary open-angle glaucoma) patients: best corrected visual acuity (BCVA), disease stage (classified after Jonas), retinal nerve fiber layer thickness (RNFL), mean defect (MD), loss variance (LV), and maximum IOP (IOP_max_).

	**BCVA**	**Stage of disease**	**RNFL [μm]**	**MD [dB]**	**LV [dB^**2**^]**	**IOP_**max**_ [mm Hg]**
OHT	0.93 ± 0.1	–	93.5 ± 16	−0.05 ± 1	2.5 ± 1.2	19.9 ± 4
Pre-POAG	0.95 ± 0.2	1.5 ± 0.9	78.5 ± 16	−0.05 ± 2	4.3 ± 3.7	19.0 ± 6
POAG	0.77 ± 0.2	2.3 ± 1.0	80.5 ± 98	7.9 ± 7	38.9 ± 26.4	17.8 ± 5
SOAG	0.65 ± 0.3	2.8 ± 1.0	60.6 ± 18	12.8 ± 8	50.5 ± 28.1	18.0 ± 4

*None of the glaucoma follow-up parameters correlated significantly with the activity of the agAAb (p > 0.05)*.

## Discussion

As shown in a previous study, pathophysiology of glaucoma is multifactorial and includes immunological components (1). Agonistic β2-agAAb were to be observed in sera of patients with OHT and POAG. Transient removal of circulating IgG including the agAAb transiently reduced the IOP, suggesting a link between β2-agAAb and IOP. As a cardiomyocyte bioassay detects the presence and function of autoantibodies, this method was used with the focus of showing functional active β2-agAAb. In the present study, we screened sera of glaucoma suspects and patients with primary and secondary glaucoma for the presence of functional active β2-agAAb. A percentage of 82 of POAG patients, and 91 of SOAG patients showed these β2-agAAb, whereas all healthy donors were seronegative. As β2-agAAb were also to be detected in sera of pre-perimetric POAG, and OHT patients, β2-agAAb, we assume that β2-agAAb are involved in the early etiology of glaucoma by a shared mechanism.

Immunological mechanisms were hypothesized to operate in glaucoma disease by several studies (17–19). However, the exact pathomechanisms of this neurodegenerative disease are still elusive. An elevated, not regulated IOP is known to be the major risk factor for onset and progression of glaucoma. Yet, most of patients with glaucoma show a disease progression despite optimal conservative and surgical IOP lowering therapies (5). Thus, several other factors contribute to its multifactorial pathogenesis (e.g., oxidative stress, vascular dysregulation) (20, 21).

It is supposed that agonistic β2-agAAb influence IOP (1), as therapy with β-blockers is common in clinical praxis. Increased IOP is caused by an imbalance between production and outflow of AH, both influenced by β2-agAAb. Experimental data showed that catecholamines can increase AH flow (22). Epinephrine induced AH flow, especially at nighttime (day: 15%: night 47%) (23). The molecular target for these clinical observations can be seen in the activity, expression and regulation of the Na-K-Cl co-transporter. As isoproterenol, a non-selective βAR agonist, is able to stimulate chloride transport via the ciliary epithelial layer (24) and Na-K-Cl co-transporter activity in pigmented ciliary body (25), it is justified to assume an influence of adrenergic agonists on the Na-K-Cl cotransporter in the ciliary body.

Considering the adrenergic subtype, application of a specific β2-blocker (ICI 118.551) diminished the stimulation of the Na-K-Cl co-transporter. We hypothesize that β2-agAAb hyperactivate the Na-K-Cl co-transporter in the ciliary body, as a similar effect of epinephrine on Na-K-Cl co-transporter activity was reported and this stimulation was inhibited by blocking the β2AR (25). The Na-K-Cl co-transporter activity then causes an increased AH flow especially at nighttime. This hypothesis is supported by clinical data of increased AH flow in OAG patients compared to normal subjects during the night hours (26). The Na-K-Cl co-transporter of the trabecular meshwork (TM), the main outflow pathway of AH, is a further potential target of the β2-agAAb which expose βAR predominantly of β2-subtype (7, 27, 28). Changes in the pore sizes of the TM, and thus of the AH outflow, were influenced by swelling and shrinkage of TM cells. The Na-K-Cl co-transporter is supposed to be a major regulator of the cellular volume and monolayer permeability regulation of TM cells, via ion transport (29). Inhibition and stimulation of the Na-K-Cl co-transporter in human TM cells leads to a reduction and increase of the volumes of TM cells, respectively (29). In cultured glaucomatous TM cells the cellular volume was reportedly enlarged when compared with healthy tissues and is reduced after incubation with bumetanide, a blocker of the Na-K-Cl co-transporter (30). Consistent with this finding the inhibition of the Na-K-Cl-co-transporter in human TM cells also led to an increased AH outflow facility (31). We hypothesize that β2-agAAb stimulate the Na-K-Cl-co-transporter in the TM cells, increase the TM cell volume with a consecutive decrease of extracellular space and outflow. This elevates the IOP.

β2AR are also present on pericytes and thus β2-agAAb may also influence the vascular microcirculation. Activation of β2AR on retinal blood vessels reportedly induce vasodilatation *in vivo* (32). A “direct” autonomic innervation is not present in retinal blood vessels (33). Yet, sympathetic activation can activate β2AR “indirectly” via its transmitter adrenaline (34). Especially, β2- and β3AR agonists have been shown to mediate retinal vasodilatation (35). According to the current data available in literature, β2AR mediate vasodilation and regulate the retinal microcirculation. We hypothesize that β2-agAAb mediated changes in retinal microcirculation further contributes to onset and progression of glaucoma, as blood flow in the optic nerve head and choroid were reduced in patients with POAG and OHT (36).

β2AR of human astrocytes and neurons of the optic nerve (9) are a third candidate target of β2-agAAb. After optic nerve transection in rabbits and rats, β2AR expression increased and peaked 90 days after intervention (9). A regulative function of astrocyte-borne β2AR has been assumed, as immature astrocytes suppressed glial scar formation (9, 37). Further support for this hypothesis was provided by an *in vivo* study on neuronal damage showing a reduced astrocyte hypertrophy and consecutive glial scar formation after application of βAR antagonists (38). Recent data showed that just an elevated IOP induced oxidative stress (via reduction in glutathione), axon degeneration of the optic nerve head and autophagy in the retinas. Thus, hypoxic glial cells can be detected in animals with OHT, even in the absence of a glaucomatous pathology (39). β2AR of astrocytes were also involved in the regulation of the glucose metabolism (40, 41) and are discussed to contribute to neuronal degeneration (42, 43).

In summary, β2-agAAb influence three factors of onset and progression of glaucoma: IOP, retinochoridal microcirculation as well as astrocyte and neuronal degeneration. All these targets are common findings in patients with OHT and POAG. Thus, β2-agAAb seemed to be a very early factor in the etiopathogenesis of glaucoma disease. Autoantibodies are hallmarks of a plethora of chronic diseases, like systemic lupus erythematosus (SLE) (44), refractory hypertension (45), type 2 diabetes (46), and dementia (15). Agonistic autoantibodies usually belong to the IgG subclasses 1–3. They are characterized by their binding to the extracellular loop 1 or 2 of neighboring G protein-coupled receptor molecules (GPCRs). This homodimerization non-canonically activates them in a manner similar to their natural ligands (47).

In contrast to the short-term activating canonical agonists, the binding of the agAAb delivers a rather stable signal, as observed in cell culture with neonatal rat cardiomyocytes. The uncontrolled, prolonged activation of the GPCR and its pathophysiological consequences was intensively investigated, especially for the AR. In a rat model, the cardiotoxic effects of agAAb against ß1AR have been demonstrated (48). Accordingly, fragments from the loop 2 of the β1AR induce dilated cardiomyopathy (DCM), also frequently associated with these agAAb in humans. In most autoimmune diseases, the pathogenic contribution of the agAAb is still elusive. Relatively clear clinical findings are present in DCM. Therapeutic apheresis (immunoadsorption) not only removed agAAb against the ß1AR from the bloodstream but also improves cardiac output for months (49). The main therapeutic target in glaucoma is the elevated IOP. Reduction of each single mmHg delays glaucoma onset and/or progression (50). Previously we reported that unspecific adsorption of IgG was accompanied by a transient decrease of IOP in patients, refractory to maximal conservative therapies (1). We argue that a specific elimination of β2-agAAb is a candidate option for the therapy of medical refractory glaucoma.

Glaucoma can be detected in up to 13% of patients with SLE. This may be caused by (I) a general predisposition to autoimmunity, (II) the interaction of the various AAb and agAAb, and (III) the therapeutic administration of glucocorticoids (51, 52). It is of interest to analyze the consequences of the interaction of different agAAb being simultaneously present in a patient.

Functional active agonistic autoantibodies directed against the GPCR are measured by using a cardiomyocyte bioassay, yet alternatives failed until now. However, western blot, immune precipitation and ELISA were unable to discriminate between agonistic autoantibodies and those that merely bind the autoantigene. None of the assays, including ours (unpublished) were able to reliably identify the agonistic antibodies. This might be due to the potential different conformation of human recombinant receptors (β2AR) compared to the highly steric specific epitope, binding agAAb. Additionally, the molecular weight of the antibody binding loop peptide (instead of the whole ß2AR) is too low for precipitation.

## Conclusion

Immunologic factors are increasingly being discussed to be involved in the etiopathogenesis of glaucoma (1, 17). Here we present data on β2-agAAb seropositivity in patients with POAG, SOAG, pre-perimetric POAG, and OHT and argue for a critical role of β2-agAAb already in an early asymptomatic phase of glaucoma disease. Further studies are justified to clarify the molecular basis of the β2-agAAb in glaucoma disease and therapy.

## Data Availability

The raw data supporting the conclusions of this manuscript will be made available by the authors, without undue reservation, to any qualified researcher.

## Ethics Statement

This study was carried out in accordance with the recommendations of Declaration of Helsinki and ethic committee of the university of Erlangen with written informed consent from all subjects. All subjects gave written informed consent in accordance with the Declaration of Helsinki. The protocol was approved by the ethic committee of the University of Erlangen.

## Author Contributions

BH, RK, MH, and GW had the idea and planned the study. US-S planned the study. GW performed the laboratory work. RL, CM, LM, SH, and FH performed and acquired the clinical trial. BH performed data acquisition and statistical analysis. BH and KK were responsible for the draft of the manuscript.

### Conflict of Interest Statement

GW, MH, and RK do have a patent (EP1832600A1). The remaining authors declare that the research was conducted in the absence of any commercial or financial relationships that could be construed as a potential conflict of interest.
